# Coping strategies of intensive care units nurses in alarm management: a qualitative research study

**DOI:** 10.1186/s12912-024-02374-1

**Published:** 2024-10-03

**Authors:** Shu-Fen Lu, Yi-Wen Kuo, Shih-Hsin Hung, Cheng-Hsueh Wu, Chien-Ying Wang, Shin-Shang Chou, Shu-He Huang

**Affiliations:** 1 Department of Nursing, Taipei Veterans General Hospital, Taipei, Taiwan, ROC; 2 Department of Nursing, Chang Jung Christian University, Tainan, Taiwan, ROC; 3 Department of Critical Care Medicine, Taipei Veterans General Hospital, Taipei, Taiwan, ROC; 4 School of Medicine, National Yang Ming Chiao Tung University, Taipei, Taiwan, ROC; 5 Department of Exercise and Health Sciences, University of Taipei, Taipei, Taiwan, ROC; 6 Vice Superintendent Office, Taipei Municipal Gan-Dau Hospital (Managed by Taipei Veterans General Hospital), Taipei, Taiwan, ROC; 7 Department of Nursing, College of Nursing, National Yang Ming Chiao Tung University, No. 12, Ln. 225, Zhixing Rd., Beitou Dist., Taipei City, 112020 Taiwan ROC

**Keywords:** Intensive care nursing, Alarm management, Alarm fatigue, Qualitative research, Coping strategies

## Abstract

**Background:**

Intensive care units are critical environments where various alarm systems play a pivotal role in patient monitoring and safety. Alarm fatigue can lead to slower response times and missed alarms, compromising patient safety and increasing stress and burnout among intensive care unit nurses. Understanding how intensive care unit nurses respond to and manage these alarms is crucial in evaluating their impact on patient care and nursing well-being.

**Methods:**

This descriptive qualitative study explored the experiences of intensive care unit nurses in alarm management. Conducted in the medical and surgical intensive care units of a Northern Taiwan medical center, the study involved 15 nurses. Semi-structured interviews were utilized to investigate the working experiences of ICU nurses in alarm management and to identify their coping strategies for dealing with the constant inundation of medical device alarms. The interviews were transcribed, and content analysis was applied to identify key themes in the responses.

**Results:**

The study revealed five main themes in intensive care unit nurses’ strategies for managing alarms: (1) Mastering alarm signals and acting; (2) Team monitoring for life preservation; (3) Enhancing senses and distinguishing carefully; (4) Learning from the lessons of incidents for vigilant reflection; and (5) Detach alarms’ influence on daily life. These coping strategies are effective in alarm management, safeguarding patients’ lives, enhancing the serenity of the clinical environment, and mitigating the physical and mental exhaustion caused by alarm fatigue.

**Conclusions:**

Intensive Care Unit nurses develop various coping strategies to manage medical device alarms, based on their experience. These strategies are crucial in maintaining patient safety and reducing nurse alarm fatigue. They can also be used for nursing education and clinical training.

## Background

Alarm fatigue is defined as healthcare providers responding to many repeated or simultaneous alarms, leading to desensitization and decreased alarm responsiveness [1]. Sensory overload occurs when clinicians are exposed to excessive alarms, which can compromise patient safety [2, 3]. Intensive Care Units (ICUs) are specialized hospital units that provide high-level care for critically ill patients. These units are equipped with sophisticated devices, equipment, and alarm systems that constantly monitor patients for changes in their physical condition, especially in life support and monitoring devices [4]. Even though medical staff are present in ICUs 24 h a day, nurses play a crucial role in recognizing and responding to clinical alarms [5].

ICU alarms are divided into two categories: non-actionable and actionable. Non-actionable alarms are temporary signals such as low oxygen or heart rate changes, which usually return to normal within seconds [3]. However, actionable alarms require immediate attention and often indicate life-threatening emergencies such as cardiac or respiratory arrest. Studies have shown that patients experience an average of 950–987 daily alarm events, translating to approximately 39.5–41.1 alarms per hour. Of these, 70.8% were valid, and 15.3% were false alarms [6]. Another study indicated that only 16.9% of monitoring alarms were properly triggered to identify a change in a patient’s status or a problem with a machine. On the other hand, 43.6% of these alerts were triggered by false positives, and 44.2% were triggered by patient movement or healthcare provider actions [7], and 85–99% of alarm do not require clinical intervention [8]. In addition, according to WHO guidelines, the ICU noise levels should be maintained between 35 and 45 dB [9]. However, frequent and varied alarms contributed to the ICU noise levels reaching 80 dB, averaging 55–60 decibels. Therefore, this noise is often perceived as “annoying” [6, 10].

ICU nurses receive extensive training, both in a classroom and through hands-on experience, to care for critically ill patients. They closely monitor patients’ physical condition and provide life support care [5]. Nurses also play a crucial role as the first responders to alarms, essential for ensuring patient safety [6]. However, a study in Korea showed that nurses experienced moderate to high levels of alarm fatigue, with scores averaging 29.1 out of 40 [6], and this contributes to alarm fatigue, loss of trust in alarms, and desensitization [3, 11]. Frequent false alarms can result in reduced attention and slower response times. Valid alarms are typically addressed within an average of 8 min, while false alarms can take up to 14 min to resolve. Additionally, when nurses misjudge non-urgent or false alarms [12], they may respond late or fail to take action, which could increase the risk of missing critical patient abnormalities and compromise patient safety [13].

Alarm fatigue is a serious issue that threatens patient safety in the digital era of medical technology. However, the work environment of ICU nurses can be overwhelming owing to the constant noise of alarms; prolonged exposure to frequent alarms disrupts nursing work. It leads to stress, tension, anxiety, and increased alarm fatigue [2] and this can have adverse health outcomes for both patients and caregivers [14]. Prominent healthcare institutions and societies in the United States have ranked alarm hazards as the top health technology concern, with the American Association of Critical-Care Nurses prioritizing them for patient safety [15]. Thus, enhancing nurses’ awareness and ability to manage alarms is crucial for reducing alarm fatigue and risks, thereby improving patient safety [4, 16].

Despite extensive research in this area, there are still significant gaps in our understanding of ICU nurses’ experiences with alarm fatigue. While recent studies have looked into specific actions and alarm customization [17], they have not fully explored how these findings apply to diverse cultural and healthcare settings [18]. Additionally, some studies provide insight into alarm frequencies and nurses’ perceptions but lack depth in understanding the clinical reasoning and decision-making processes influencing nurses’ response to alarms [6, 11]. One study on Iranian ICU nurses highlighted that alarm fatigue threatens personal balance, leading to coping strategies such as ‘smart care,’ ‘deliberate balancing,’ ‘conditional prioritization,’ and sometimes ‘negligent performance‘ [19]. However, these strategies are significantly impacted by broader organizational issues such as staff shortages and inadequate infrastructure [14]. Another study found systemic factors like organizational support and alarm system design are crucial in shaping how nurses respond to alarms [20]. Overall, there is a need for a more comprehensive approach to address individual coping strategies and systemic issues within the ICU environment. Therefore, it is crucial to develop coping strategies to assist ICU nurses in addressing alarm management challenges, enhancing the quality of care for critically ill patients, and improving their working conditions. This study aims to explicitly investigate ICU nurses’ management strategies and response modes to alarms. By examining the experience and wisdom of ICU nurses in coping strategies and approaches to effectively respond to various alarm situations in the intensive care unit. The insights gained from this study may prove beneficial for others seeking to reduce ICU alarm fatigue, enhance patient care, and improve the working lives of nurses.

### Aim

This study investigated ICU nurses’ management strategies and response modes to alarms. By examining the perspectives of ICU nurses, we explored their coping strategies and approaches to effectively respond to various alarm situations in the intensive care unit.

## Materials and methods

### Study design

This study used a descriptive qualitative approach to explore the experiences of ICU nurses in managing alarms. This approach was chosen due to its suitability in systematically analyzing and interpreting the experiences of nurses in alarm management. The qualitative content analysis method was particularly effective in extracting key themes from the semi-structured interview data, which is highly aligned with the study’s goal of uncovering the strategies and challenges nurses face when dealing with alarm systems. Qualitative content analysis provides the necessary flexibility to systematically categorize data while deeply understanding the underlying meanings. Verbatim transcriptions of the interviews were subjected to content analysis to identify key themes and patterns. Compared to other methods, such as phenomenology or grounded theory, qualitative content analysis is more appropriate for describing nurses’ wisdom acquired by learning from the working experiences with alarm management rather than generating new theories or focusing on deeply personal experiences. In addition, the research design and data analysis team, consisting of ICU doctors, nurses, and nursing researchers, brings extensive expertise to the study design, data analysis, and context.

### Setting and participants

This research was conducted in a 42-bed medical and surgical ICU at a medical center in North Taiwan from January 16 to December 28, 2018. The ICU operates on a three-shift system and maintains a nurse-to-patient ratio of approximately 1:2.6. It employs a primary nursing model, enhancing personalized and consistent patient care. Advanced monitoring systems such as Philips IntelliSpace Critical Care and Anesthesia (ICCA), Philips Central Monitoring Alarms, and a Nursing Information System (NIS) are utilized to manage and document patient care.

Additionally, the ICU has established a structured preceptorship program for new nurses. These nurses typically have completed a two-year post-graduate clinical training and must undergo a three-week lecture course and clinical practicum before commencing clinical work in the ICU. This training program is tailored to meet the institution’s critical care quality; the course includes the medical device alarm management standards, ensuring that new nurses are well-prepared to handle the complexities of caring for critically ill patients in the ICU environment.

Participants were selected through purposive sampling to ensure a diverse representation of ICU nurses’ experiences with alarm management. The inclusion criteria required participants to be ICU nurses with at least six months of experience working in the ICU and to consent to participate in the study. Exclusion criteria excluded nurses undergoing short-term training or those in support roles. The recruitment process involved voluntary participation, and the second author obtained informed consent from each participant before data collection. Several measures were implemented to address potential biases. We selected nurses with different shift schedules, varying levels of ICU experience, including both frontline staff and nursing team leaders, to minimize selection bias and capture diverse perspectives on alarm management, reflecting a broader ICU nursing population. A total of 15 ICU nurses were recruited for the study, and no participants withdrew. The sample size in qualitative research was determined based on the concept’s guiding principle of “data saturation” [21].

### Data collection

This study followed established guidelines for conducting interviews. Before the formal interviews, a pilot interview was conducted using interview guidelines to refine interviewing techniques and content. To address interviewer bias, the second author, with 17 years of clinical intensive care nursing and qualitative research experience, conducted semi-structured interviews with two ICU nurses to gather advice on arranging and revising the content of the interview guidelines and improving the interview skills, ensuring that the interviews were conducted in a consistent and unbiased manner. Subsequently, the research teams discussed and revised the interview guidelines accordingly. The final interview guidelines are as follows:

1. How frequently do you encounter machine alarms at your workplace?
2. In what ways do these alarms affect you at work?
3. Do you think that you may become desensitized to sound due to the frequent exposure to these alarms?
4. Do these alarms have a lasting impact on your body, mood, or personal life after work?
5. What are your typical strategies for minimizing or mitigating the effects of these alarms on your work or personal life?
6. Can you share a specific experience where not responding to a machine alarm at work made a strong impression on you? How did this experience shape your approach to managing alarms?

During the initial phase, the first author elucidated the study’s purpose, invited participants to participate in research interviews, and affirmed their freedom to participate. The second author then expounded on the study’s objectives, confidentiality assurances, and the procedure for obtaining participants’ informed consent. Subsequently, the second author commenced the semi-structured interviews only after the informed consent was secured. These one-on-one interviews, conducted in a private ICU room, lasted an average of 50 min to one hour and were audio recorded for accuracy. The researcher maintained an open-minded and empathetic demeanor, actively listening to the participants’ narratives. The interview process included follow-up inquiries to validate the information provided. A total of 15 interviews were conducted without any repeats, and field notes were taken to capture additional observations.

### Data analysis

This research used qualitative content analysis for data analysis; data are presented in words and themes, which makes it possible to draw some interpretation of the results for ICU nurses’ alarm management. The coding process was conducted manually to ensure a deep and thorough engagement with the data. Two data coders, Yi-Wen Kuo and Shu-Fen Lu, independently coded the data. To ensure inter-coder reliability, the coders compared their initial coding results and resolved any discrepancies through discussion and consensus. The final coding was then reviewed with the research team to ensure accuracy and agreement. The transcripts were returned to the participants for their comments and/or corrections to verify the accuracy of the content. Data saturation was achieved when no new themes emerged from the meaningful data extracted during the analysis. All research data were securely stored in a locked personal office of the researcher and protected by password-encrypted computer files to ensure confidentiality. This methodology involved carefully designed steps, as outlined by Krippendorff (2004) [22].

- The interview recordings were carefully listened to understand the participants’ experiences fully. Everything was written down exactly as it was said, giving us valuable firsthand information.
- Each sentence was reviewed and analyzed according to the principle of staying true to the original intent. Descriptive statements relevant to the clinical research questions were identified.
- The data identified meaningful sentences that accurately represented the original intent.
- Descriptive sentences with significant relevance were logically grouped to form “descriptive characteristics,” which combined common characteristics to create preliminary themes.
- A composite description was created for each of the preliminary themes.
- Common “descriptive characteristics” from each case were categorized into common themes guided by the preliminary themes. This helped to shape the essence of the phenomenon.
- The interviewees reviewed the results to ensure the findings accurately reflected the nursing staff’s experiences. Additional data provided by participants were incorporated into the final structure.

### Trustworthiness

The reliability and validity of this study are anchored in the rigor of qualitative research methodologies, adhering to the standards set forth by Lincoln and Guba (1985) [23]: credibility, transferability, dependability, and confirmability.

The study’s credibility was established through the researcher’s extensive experience as an ICU nurse, which enabled a deep connection with the subjects. This rapport facilitated an environment where the participants openly shared their experiences and feelings regarding alarm alerts. The researcher’s empathy allowed for a genuine understanding of their experiences, further enriched by continuous observation of their alarm management behavior and emotional responses in the workplace.

Regarding transferability, the study employed purposive sampling to select nursing staff with at least 6 months to 16 years of ICU experience. This approach ensured a rich diversity of experiences, enhancing the applicability of the study to similar phenomena in other ICUs. Data collection was characterized by a non-judgmental and stream-of-consciousness approach during interviews, complemented by interactive observations. This comprehensive data collection strategy contributed to the richness and applicability of the findings.

The study’s dependability was ensured by including verbal and nonverbal data in the analysis. The researcher carefully recorded audio and interview data, highlighting the participants’ perspectives through a comprehensive, step-by-step content analysis. Multiple discussions and reviews were conducted with a group of experts in qualitative research with similar care backgrounds to ensure the accuracy of the analysis. These discussions facilitated a consensus on categories, subthemes, and themes, with constant checking and revision to ensure that everything was noticed.

Finally, confirmability was addressed by maintaining a thorough record of all data, including tapes, verbatim texts, interview records, observations, reflections, and the entire data analysis process. This extensive documentation was coded, categorized, and adequately archived, allowing for future inspection and verification and ensuring the study results were repeatable and verifiable.

### Ethical considerations

The study was approved by the hospital’s Institutional Review Board of Taipei Veterans General Hospital (IRB No: 2017-07-032 C). Participants were fully informed about the study’s purpose, procedures, and their voluntary participation. They provided informed consent and were guaranteed confidentiality throughout the research process, with each participant assigned a numeric code to ensure anonymity. Ethical considerations were central to the study, guiding the methodology to minimize harm. Confidentiality was strictly maintained, with participants able to review and amend their transcripts. To reduce stress, interviews were conducted in a supportive, non-judgmental environment, encouraging open and honest discussions.

## Results

The demographic characteristics of the 15 interviewed nurses are detailed in Table 1. The participant’s ages ranged from 27 to 46 years, and 14 of them were female. Years of experience as a Registered Nurse (RN) varied from 6 to 25 years, and critical Unit (CU) experience ranges from 0.5 to 16 years.

**Table 1:** Demographic characteristics (*N* = 15)

Code	Age	Sex	Education	RN experience (Years)	CU experience (Years)	Marital status
A	35	Female	Bachelor	14	10	Single
B	32	Female	Bachelor	12	5	Single
D	28	Female	Master	6	3	Single
E	36	Female	Bachelor	16	8	Married
F	43	Female	Bachelor	23	13	Single
G	42	Female	Bachelor	20	11	Single
H	34	Female	Bachelor	12	3	Single
I	43	Female	Bachelor	23	7	Single
J	35	Female	Bachelor	14	7	Single
K	45	Female	Bachelor	25	16	Married
L	30	Female	Bachelor	7	0.9	Single
M	38	Female	Bachelor	15	1.2	Married
N	35	Female	Bachelor	14	6	Married
O	27	Female	Bachelor	6	0.5	Single
P	46	Male	Master	22	10	Married

### Core themes

**Fig. 1:**
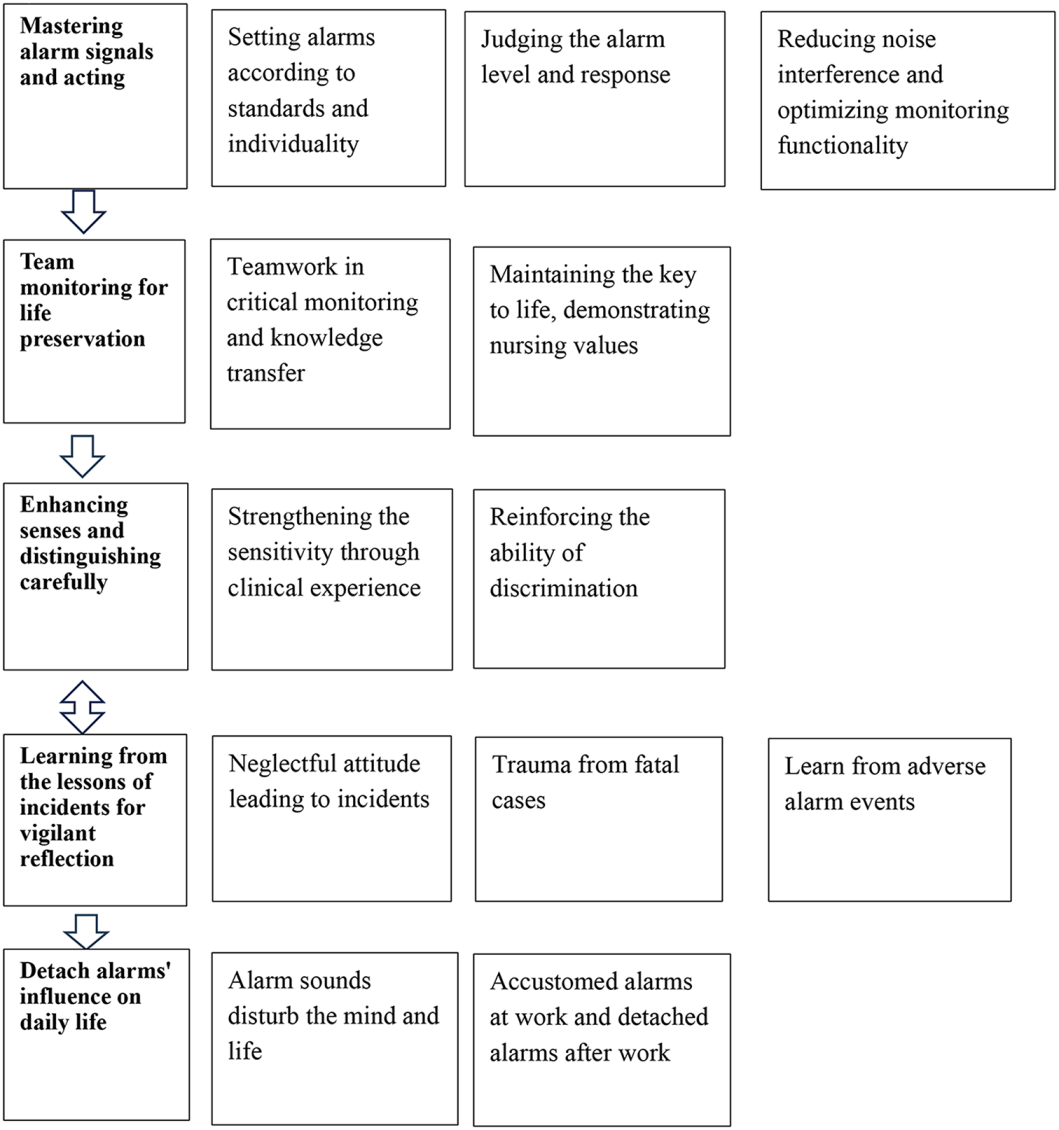
Coping strategies of intensive care units nurses in alarm management

Five themes emerged from the analysis: (1). Mastering alarm signals and acting, (2). Team monitoring, for life preservation, (3). Enhancing senses and distinguishing carefully, (4). Learning from the lessons of incidents for vigilant reflection, and (5). Detach alarms’ influence on daily life. Each theme represents the experience of ICU nurses’ alarm management and coping strategies, as shown in Fig. 1. Table 2 provides an example of the process of data analysis and processing.

**Table 2:** Example of final themes, sub-themes, and interview transcript

Theme	Sub-theme	Interview transcript
Mastering alarm signals and acting	Setting alarms according to standards and individuality	If you do not have experience with a patient, you will not know the appropriate settings for them. …When I first started, I just followed the standards. As I gained experience, I realized that I should adjust the settings a bit broader for some stable patients, and the patient’s condition is critically dangerous; I would not… set it too broadly!
	Judging the alarm level and response	The patient is everyone’s responsibility. Red alarms are an emergency… a patient might face a life-threatening situation. We rush over. Our role is to discover their condition immediately!
	Reducing noise interference and optimizing monitoring functionality	You learn to adjust and trust your judgment. …If the alarm rings too often, you might ignore it but reset it later. If it keeps ringing outside the adjusted range, there might be something wrong with the patient.
Team monitoring for life preservation	Teamwork in critical monitoring and knowledge transfer	In the ICU, teamwork is crucial. Whenever there’s an emergency alarm, we all rush to help. As senior members, we should teach and support our juniors rather than scolding them. However, as leaders, we must strike the right balance to ensure that our juniors are not afraid to respond to the alarm.
	Maintaining the key to life, demonstrating nursing values	ICU alarms are like lifelines. The EKG machine has five lines; if one goes awry, it feels like a lifeline being severed. Quick resolution is crucial to prevent losing a vital lifeline for the patient.
		As a nurse, I am always on high alert at work because timely detection and treatment of patients are critical to their well-being.
Enhancing senses and distinguishing carefully	Strengthening the sensitivity through clinical experience	At first, I got nervous hearing alarms. I couldn’t identify the direction or which bed was alarming. My response felt slow.
		I was nervous the first time I operated an ECMO machine, and it kept alarming. A senior nurse explained everything, and I became more careful in avoiding negligence.
	Reinforcing the ability of discrimination	At work, you must keep your ears open. After starting work, I became more sensitive to sounds… It is a concept you must address, not just complain about how annoying or noisy it is. It is a skill developed through work training.
Learning from the lessons of incidents for vigilant reflection	Neglectful attitude leading to incidents	Ventilator alarm rang while I was bathing a patient. The nurse assigned to the patient was busy with paperwork and did not hear the alarm. I looked at her and saw that she had assumed the alarm was not from her patient.
	Trauma from fatal cases	A patient self-extubated and bled heavily… If the response had been quicker, maybe he would not have died. Everyone seems insensitive to alarms. Now, I feel tension and am always on alert for every alarm… It is a huge responsibility!
	Learn from adverse alarm events	After a patient removed the endo tube and triggered the ventilator alarm, Unfortunately, the patient died. I realized the importance of taking proactive measures. Now, I am more vigilant in my work and frequently check on a patient’s status after adjusting their position to prevent unexpected incidents. It’s all about avoiding regrets.
Detach alarms’ influence on daily life	Alarm sounds disturb the mind and life	Alarm sounds? Irritating.
		Sometimes, emotions follow me home; I have nightmares, tension, and panic. Work affects family and social life.
	Accustomed alarms at work and detached alarms after work	No matter what, it is just these 8 h. After work, it does not affect my life. I’ve completely adapted… These sounds disappear once I leave work, completely detached.

#### Mastering warning signals and acting

The ICU nurses have been trained to distinguish between different alarm sounds that indicate the urgency of a situation. Hearing frequent alarms from monitoring devices can be disruptive and may cause delays in response. However, with experience, nurses learn to constantly observe, adapt, evaluate, to ensure that alarm sounds and management effectively serve their intended purposes. This coping strategy includes three sub-themes: (1) setting alarms according to standards and individuality; (2) judging the alarm level and response; and (3) reducing noise interference and optimizing monitoring functionality.

Each of these aspects is explained in detail below.

##### Setting alarms according to standards and individuality

Nurses typically begin with standard alarm settings, but as they gain experience, they adjust based on the patient’ condition. They recognize that rigid adherence to these standard protocols can result in a constant stream of alarms triggered by regular changes in a patient’s condition, making it difficult to assess the severity of each situation. As a result, nurses learn to tailor alarm settings to the patient’s condition to improve monitoring effectiveness. They expand alarm thresholds for stable patients and carefully adjust them for critically ill patients, ensuring timely emergency response. This approach enhances monitoring effectiveness by broadening alarm thresholds for stable patients and making precise adjustments for critically ill patients.

This indicates how nurses adapt and modify their approach as they gain experience, balancing the need for vigilance with the practicalities of alarm fatigue. The ability to make these adjustments reflects a deeper understanding of patient needs and alarm systems, which is essential for effective ICU care.

*Nurse J: “If you do not have experience with a patient*,* you will not know the appropriate settings for them. …When I first started*,* I just followed the standards. As I gained experience*,* I realized that I should adjust the settings a bit broader for some stable patients*,* and the patient’s condition is critically dangerous; I would not… set it too broadly!”*

##### Judging the alarm level and response

In ICUs, medical devices produce alarms that indicate the urgency of a patient’s condition. These alarms are associated with different sounds, and nurses determine the speed of their responses based on the urgency of the alarm. This process involves identifying the cause of the alarm and ensuring a timely response to maintain the patient’s life.

The differentiation between alarm types and the corresponding actions taken by nurses demonstrate their critical thinking and decision-making abilities, which are vital in high-pressure environments like the ICU.

*Nurse C: “The patient is everyone’s responsibility. Red alarms are an emergency… a patient might face a life-threatening situation. We rush over. Our role is to discover their condition immediately!”*

##### Reducing noise interference and optimizing monitoring functionality

ICU alarms can overwhelm nurses, leading to potentially missed warnings. Nurses assessed interference, identified causes, and adjusted settings to optimize alarm functionality and ensure.

This illustrates the importance of environmental management in the ICU, where reducing unnecessary noise and interference can enhance the clarity and effectiveness of critical alarms, thereby improving patient outcomes.

*Nurse H: “*Y*ou learn to adjust and trust your judgment. …If the alarm rings too often*,* you might ignore it but reset it later. If it keeps ringing outside the adjusted range*,* there might be something wrong with the patient.”*

#### Team monitoring for life preservation

In the ICU, alarms play multiple vital roles. They act as predictions, reminders, and aids in patient care. It’s crucial to carefully address each alarm the patient’s life. Team leaders are responsible for monitoring all patient alarms and guiding the team in responding appropriately to different alarms. They also empower less-experienced nurses to gain valuable experience handling alarms, enhancing their professional competence and ensuring that the entire team collectively maintains patient safety. This theme has two sub-themes: (1) Teamwork in critical monitoring and knowledge transfer and (2) Maintaining the key to life and demonstrating nursing value.

##### Teamwork in critical monitoring and knowledge transfer

ICU leaders monitor and respond to life-threatening alerts. They assist less-experienced colleagues who need more sensitivity to these alerts and remind senior nurses to prevent missing critical warnings. Leaders prioritize the urgency of alerts, providing opportunities for colleagues to handle less critical situations independently while personally addressing critical alarms. This approach ensures team cohesion, confidence in following the guidance, and patient safety.

*Nurse F: “In the ICU*,* teamwork is crucial. Whenever there’s an emergency alarm*,* we all rush to help. As senior members*,* we should teach and support our juniors rather than scolding them. However*,* as leaders*,* we must strike the right balance to ensure that our juniors are not afraid to respond to the alarm.”*

##### Maintaining the key to life, demonstrating nursing values

Monitoring devices in the ICU emit sounds that demand attention, serving as vital indicators of patient status. Overlooking these alarms can result in omitting critical changes in a patient’s condition. Nurses seriously heed every warning to guarantee the safety and well-being of their patients.

*Nurse G: “ICU alarms are like lifelines. The EKG machine has five lines; if one goes awry*,* it feels like a lifeline being severed. Quick resolution is crucial to prevent losing a vital lifeline for the patient. “*.

*Nurse F believed: “As a nurse*,* I am always on high alert at work because timely detection and treatment of patients are critical to their well-being.”*

#### Enhancing senses and distinguishing carefully

The nurses’ ability to handle alarms depends on their senses. With experience, they develop sharp auditory responses and situational awareness. They prioritized discerning the urgency of each alarm to comprehend the patients’ conditions promptly. This theme included two subthemes: (1) strengthening the sensitivity through clinical experience and (2) reinforcing the ability of discrimination.

##### Strengthening the sensitivity through clinical experience

New ICU nurses with limited experience and less sensitivity, may struggle to differentiate between different alarm sounds, their sources, and their meanings. However, as they gain more experience, they learn to approach alarms cautiously and seek guidance from experienced colleagues. Reflecting on their experiences helps them develop skills and knowledge, which ultimately allows them to prevent adverse events.

*Nurse H recalled: “At first*,* I got nervous hearing alarms. I couldn’t identify the direction or which bed was alarming. My response felt slow.”*.

*Nurse D shared: “I was nervous the first time I operated an ECMO machine*,* and it kept alarming. A senior nurse explained everything*,* and I became more careful in avoiding negligence.”*

##### Reinforcing the ability of discrimination

The alarms from medical devices are crucial signals for ensuring patient safety in the ICU. However, it can be challenging for nurses, especially newly trained ones, to recognize and respond to these alerts in a complex environment where many alarms may be sounding simultaneously. Experienced nurses are more skilled at identifying the source of an alarm and interpreting the meaning of a patient’s status.

*Nurse E: “At work*,* you must keep your ears open. After starting work*,* I became more sensitive to sounds… It is a concept you must address*,* not just complain about how annoying or noisy it is. It is a skill developed through work training.”*

#### Learning from the lessons of incidents for vigilant reflection

Taking all alarms seriously is crucial, as a slight delay can lead to difficult-to-remedy injuries Ignoring alarms can have irreversible and unbearable. consequences. Missed alarms often occur due to underestimating the severity, lack of alertness, or failure to address the root cause. It is critical to thoroughly examine the underlying causes of incidents and remain vigilant in learning from them to prevent future occurrences. The theme includes three subthemes: (1) Neglectful attitudes leading to incidents; (2) Trauma from Fatal Cases, (3) Learn from adverse alarm events.

##### Neglectful attitude leading to incidents

In the ICU, each bed is assigned a caregiver. Unfortunately, some nurses might ignore alarms if they believe they aren’t relevant to their patient’s health or don’t to be disturbed by the noise. Sometimes, they might adjust the settings based on their judgment rather than the patient’s condition or turn off the alarms.

*Nurse B: “Ventilator alarm rang while I was bathing a patient. The nurse assigned to the patient was busy with paperwork and did not hear the alarm. I confronted her and discovered she had assumed the alarm was not from her patient.”*

##### Trauma from fatal cases

Nurses who were unable to respond promptly to an alarm resulting in a patient’s passing expressed a deep sense of helplessness and remorse. They deeply regretted not acting immediately and believed that the patient’s life could have been saved. This highlights how even the most skilled, healthcare providers are affected by alarm incidents.

*Nurse A: “A patient self-extubated and bled heavily… If the response had been quicker*,* maybe he would not have died. Everyone seems insensitive to alarms. Now*,* I feel tension and am always on alert for every alarm… It is a huge responsibility!”*

##### Learn from adverse alarm events

Healthcare professionals have become more careful when verifying alarm triggers after witnessing dangerous incidents because of their insufficient experience. They urged others to remain proactive, maintain ongoing patient monitoring, and document developments, especially when dealing with unsettled or critically ill patients.

*Nurse K: “After a patient removed the endo tube and triggered the ventilator alarm*,* Unfortunately*,* the patient died. I realized the importance of taking proactive measures. Now*,* I am more vigilant in my work and frequently check on a patient’s status after adjusting their position to prevent unexpected incidents. It’s all about avoiding regrets.”*

#### Detach alarms’ influence on daily life

The ICU heavily relies on device alarms to monitor patients and ensure their well-being. However, can be disruptive, causing fatigue and stress in nurses., particularly for those who are new to the field. This stress can extend beyond work hours and affect personal lives. It is also important to note that colleagues’ reactions to alarms can impact nurses’ emotional well-being. Despite this, some nurses consciously try to mentally detach from work, to mitigate the impact on their personal lives. This theme comprises two subthemes: (1) alarm sounds disturb the mind and life, (2) accustomed alarms at work and detached alarms after work.

##### Alarm sounds disturb the mind and life

In the confined space of the ICU, the constant echoing of alarms can be overwhelming, especially for new nurses who are not yet accustomed to frequent alarms and struggle to identify their source and meaning. Failure to manage them effectively can cause anxiety, disrupted sleep, and emotional distress, all of which impact one’s personal life.

*Nurse L: “Alarm sounds? Irritating.”*.

*Nurse I: “Sometimes*,* emotions follow me home; I have nightmares*,* tension*,* and panic. Work affects family and social life. ”*.

##### Accustomed alarms at work and detached alarms after work

As nurses amass professional experience, they become accustomed to alarm sounds and can disengage from work, thereby acquiring the skill of maintaining a wholesome work-life balance.

*Nurse B: “No matter what*,* it is just these 8 hours. After work*,* it does not affect my life. I’ve completely adapted… These sounds disappear once I leave work*,* completely detached.”*

## Discussion

This study investigated ICU nurses’ management strategies and responses to various patterns of alarms in critical care settings. Our findings identified five key themes, which mainly emphasized the importance of nurses correctly identifying medical device alarms, responding rapidly, and providing professional care to monitor and maintain the health status of critically ill patients and how they accumulated professional capabilities through clinical work experience. In addition, it aims to minimize the impact of alarms on caregivers’ own lives by developing the ability to cope with alarm fatigue.

Ethical considerations were integral to the study’s design. Recruitment was managed by non-supervisory team members to ensure voluntary participation, and confidentiality was strictly maintained to avoid impacting professional evaluations. Interviews were scheduled on participants’ non-duty days to reduce stress and ensure well-being. Additionally, transparency in data results was prioritized, allowing participants to review and confirm their transcripts. These ethical measures guided the data collection and analysis, ensuring the study respected participants’ rights and produced reliable, transparent, and ethically sound findings.

The study revealed how ICU nurses manage and respond to alarms in critical care settings, highlighting the professional and ethical importance of correct and rapid identification and handling of alarms. Nurses expressed the importance of accurately identifying medical device alarms and emphasized using clinical cases to improve professional alarm management ability by enhancing sensory perception and making careful distinctions, learning from incidents and remaining vigilant, and modulating setting the alarm threshold by professional judgment to minimize the false alarm. These findings emphasized nurses’ complex and multi-layered approaches to ensuring patient safety within the ICU. This study provides a foundation for developing targeted training programs that focus on enhancing nurses’ alarm management skills, thereby improving patient outcomes and reducing the risk of alarm fatigue.

Mastering alarm signals and acting is considered a fundamental skill for ICU nurses. Nurses develop rapid response abilities to various alarms through clinical practice, supported by a deep understanding of alarm systems and patient conditions. This finding aligns with Carelli et al. [24], who emphasized the importance of mastering alarm signals to react and handle alerts instantly. Similarly, Anderson et al. [15] highlighted that timely and accurate responses to alarms are crucial for patient safety, particularly in high-critical illness care settings. However, unlike previous studies, our research highlights that this skill development is not solely dependent on technical training but is also significantly influenced by continuous experiential learning and reflection, a concept supported by Cvach [3], who discussed the importance of ongoing education and hands-on working experience in improving alarm management ability. This insight can be applied to develop more comprehensive training programs that combine technical knowledge with practical experience, leading to more effective alarm management practices in ICU settings. Additionally, recent research by Movahedi et al. (2024) supports the use of smart care systems to reduce alarm fatigue through reflective practice [18].

Alarm management extends beyond individual effort, requiring cohesive teamwork to ensure patient safety. Medical team collaboration enhances alarm management efficiency, and our findings were consistent with Anderson et al. [15], who reported that team monitoring reduces medical errors. This underscores the critical role of collaboration within the ICU, as team collaboration in monitoring for life preservation and enhancing alarm management efficiency is essential. Additionally, the study by Lopez-Espuela et al. [20] supports our findings by showing that collaborative approaches in alarm management can improve overall patient outcomes and mitigate the effects of alarm fatigue [20], which elucidates the specific mechanisms of team interaction, such as role allocation and real-time communication. Despite these, which are less explored in existing literature but critical for effective alarm management, as highlighted by Cvach et al. [10]. The findings suggest that promoting teamwork and real-time communication should be key components in alarm management strategies, which can be incorporated into ICU protocols to enhance patient safety and reduce the incidence of alarm-related errors.

The ability of alarm management accumulated through clinical experience. Enhancing sensory perception and making careful distinctions is another crucial strategy identified in high-pressure ICU environments. Our study extends this understanding by suggesting that sensory perception enhancement is largely a product of sustained practice and reflection rather than solely relying on literature education. As nurses accumulate clinical experience, they become increasingly adept at discerning subtle differences between alarm sounds, leading to more effective clinical decision-making and reactions. This finding is supported by the study finding of Ceylan et al. [5], who highlighted the core components of sensory perception in alarm management. That is also demonstrated by an observational study finding by Wang et al. [6] and further reinforced by Milhomme and Pomerleau in their integrative review that experienced nurses are better equipped to identify and prioritize critical alarms, reducing the likelihood of alarm desensitization and improving patient care accuracy [25]. The practical implication is that continuous professional development focused on sensory perception and decision-making in alarm management can enhance the effectiveness of ICU care.

In addition, it is known that ICU nurses accumulate experience in alarm management through past events. Learning from incidents and remaining vigilant reflects the continuous learning and reflection process that ICU nurses undergo in alarm management by reviewing and analyzing past alarm events. This observation aligns with the conclusions of Milhomme and Pomerleau, who identified reflective clinical practice as a key factor in improving alarm management capabilities [25]. Moreover, the study by Honan et al. emphasized that learning from critical incidents not only enhances alarm management but also contributes to the development of a safety culture within the ICU, which is essential for preventing alarm fatigue and improving patient outcomes [26]. Our study suggests that this learning process involves not only technical skill improvement but also a re-evaluation of professional roles, especially in high-risk scenarios, an aspect that is underexplored in previous research but crucial for developing comprehensive alarm management strategies. These insights highlight the need for structured reflective practices and ongoing learning opportunities for ICU nurses, which could be integrated into professional development programs to sustain high standards of patient care.

The study highlights the importance of psychological adjustment in managing the stresses of a high-pressure work environment for ICU nurses, including detaching from the influence of alarms on daily life. Experienced nurses in our study demonstrated the ability to manage stress effectively, which supports their professional sustainability and positively impacts their overall quality of life. This finding is consistent with Lopez-Espuela et al. [20], who noted the benefits of stress management strategies in mitigating the negative impacts of alarm fatigue. Similarly, the systematic review by Gul et al. [27] found that implementing stress management interventions in ICU settings significantly reduces the occurrence of alarm fatigue among nurses, leading to better mental health and improved job satisfaction [10]. Recent studies, such as those by Salameh et al. [28], have also emphasized the link between perceived stress and alarm management performance, suggesting that stress reduction may enhance nurses’ ability to respond to alarms more effectively [7, 28]. Our study adds to this by showing that the development of such coping mechanisms is rooted in years of clinical experience, reflection, and team support, an aspect that has not been thoroughly examined in earlier studies but is essential for long-term nurse well-being as discussed by Honan et al. [26]. These findings emphasize the need for structured stress management programs that integrate both psychological support and technical skill development. Such programs can promote a healthier work-life balance and improve alarm management practices in ICU settings. Implementing these initiatives would enhance nurse well-being and contribute to the delivery of high-quality patient care. In summary, this study reveals ICU nurses’ diverse strategies in alarm management, emphasizing the importance of technical skills, sensory acuity, and teamwork. These findings enrich the existing literature by integrating clinical practice with reflective processes to enhance alarm management abilities. They also provide new directions for future research, especially in optimizing alarm management strategies across different healthcare environments to improve overall care quality. By implementing the practical recommendations derived from this study, healthcare organizations can enhance alarm management practices, ultimately leading to better patient outcomes and reduced nurse burnout.

This study has several limitations. Data were collected from senior and junior nursing staff to capture a comprehensive perspective of an adult medical and surgical ICU at a medical center in Taiwan. However, it is important to acknowledging that these findings may not be generalizable across all ICU nursing domains. Firstly, the interviewers and respondents in this study were all nurses from the same unit, which might affect their ability to fully express their true opinions.

The personal attitudes and moral motivations of the respondents may have influenced their ability to accurately represent the reality of alarm handling. Additionally, external factors, such as the hospital’s alarm management policies, resource allocation (e.g., nurse-to-patient ratios, availability of technical support), and the condition of the equipment, and the ongoing impact of COVID-19 on healthcare practices, may have also impacted the study results. Differentiating between the significance of different alarms and the appropriate response is crucial, especially for raising awareness of patient safety among new and inexperienced staff in intensive care. Our findings provide practical implications for developing strategies to manage alarm systems and clinical alert care standards, ultimately in order to reduce alarm fatigue in clinical settings. These findings also offer insights for shaping nurses’ education, training, and quality management. For example, actions like adjusting alarm settings, eliminating duplicate alarms, and customizing alerts based on patient conditions can ensure that nursing staff receive effective alerts and respond promptly, reducing the risk of alarm fatigue. Based on the study findings, we recommend annual training sessions on alarm knowledge and skills, continuous device monitoring, and establishing alarm event forums to strengthen a consensus on alarm-handling standards and improve overall alarm management among clinical nurses.

To further validate and expand these findings, additional research is necessary. Future studies should explore alarm management strategies across different types of ICUs, such as pediatric, cardiac, or respiratory specialty units, and investigate practices in different geographic regions, including rural areas, regions with varying healthcare infrastructure, and teaching hospitals. Furthermore, research should explore how collaboration among different healthcare roles, such as on-duty physicians and respiratory therapists, can lead to more effective alarm management strategies, thereby improving clinical practice and enhancing patient safety. This continued research will help ensure the relevance and applicability of alarm management strategies in diverse healthcare environments, ultimately contributing to improved critically ill patient safety and intensive care unit nurses’ well-being.

## Conclusions

The ICU environment requires a more comprehensive approach to address individual coping strategies and systemic alarm fatigue issues. Thus, it is essential to develop coping strategies to assist ICU nurses in addressing alarm management challenges, improving the quality of care for critically ill patients, and enhancing their working conditions. This study aims to investigate ICU nurses’ management strategies and response modes to alarms, drawing from the experience and wisdom of ICU nurses’ effectively responding to various alarm situations in the intensive care unit. The insights gained from this study may benefit others in reducing ICU alarm fatigue, improving patient care, and enhancing the working lives of nurses. This study highlights ICU nurses’ critical role in ensuring patient safety and well-being. Five core themes emerged from the study results, representing that ICU nurses take responsibility for monitoring alarm alerts with heightened senses and accurately differentiate between various alarms. They work collaboratively, to manage alarm events and sustain patients’ lives. The findings reveal that through experience, self-reflection, and guidance from experienced staff, nurses develop awareness, judgment, and the ability to handle alarms, prioritize patient safety, and adhere to professional ethics in an ICU work environment. This study significantly contributes to theory and practice by identifying critical areas for enhancing ICU alarm management. The findings deepen our understanding of alarm management’s psychological and ethical aspects and offer practical recommendations for targeted training and support. By addressing the emotional and psychological impacts of frequent alarms, these measures will improve patient safety and nurse well-being.

## Data Availability

The datasets analysed during the current study are available from the corresponding author on reasonable request.

## Abbreviations

ICU: Intensive Care Unit
RN: Registered Nurse
ECMO: Extracorporeal Membrane Oxygenation
EKG: Electrocardiogram
